# How to explore the needs of informal caregivers of individuals with cognitive impairment in Alzheimer’s disease or related diseases? A systematic review of quantitative and qualitative studies

**DOI:** 10.1186/s12877-017-0481-9

**Published:** 2017-04-17

**Authors:** T. Novais, V. Dauphinot, P. Krolak-Salmon, C. Mouchoux

**Affiliations:** 1 EA-7425 HESPER, Health Services and Performance Research, University Lyon, F-69003 Lyon, France; 2 Clinical Research Centre (CRC) - VCF (Aging – Brain - Frailty), Charpennes Hospital, University Hospital of Lyon, F-69100 Villeurbanne, France; 3 Pharmaceutical Unit, Charpennes Hospital, University Hospital of Lyon, F-69100 Villeurbanne, France; 4 Clinical and Research Memory Centre of Lyon (CMRR), Charpennes Hospital, University Hospital of Lyon, F-69100 Villeurbanne, France; 5 University Lyon 1, F-69000 Lyon, France; 6 INSERM U1028, CNRS UMR5292, Lyon Neuroscience Research Center, Brain Dynamics and Cognition Team, University Lyon 1, F-69000 Lyon, France

**Keywords:** Caregivers, Needs assessment, Mild cognitive impairment, Dementia, Alzheimer’s disease and Related Diseases, Systematic review, Quantitative studies, Qualitative studies

## Abstract

**Background:**

This study aims to review the methodologies used to identify the needs, the existing needs assessment instruments and the main topics of needs explored among caregivers of patients with mild cognitive impairment to dementia.

**Methods:**

MEDLINE, PsycINFO, The Cochrane Library and Web of science were searched from January 1980 to January 2017. Research studies in English or French were eligible for inclusion if they fulfilled the following criteria: quantitative, qualitative and mixed method studies that used instrument, focus group or semi-structured interviews to assess the informal caregiver’s needs in terms of information, coping skills, support and service.

**Results:**

Seventy studies (*n* = 39 quantitative studies, *n* = 25 qualitative studies and *n* = 6 mixed method studies) met the inclusion criteria and were included. Thirty-six quantitative instruments were identified but only one has been validated for the needs assessment of dementia caregivers: the Carer’s Needs Assessment for Dementia (CNA-D). The main areas of needs explored in these instruments were: information, psychosocial, social, psychoeducational and other needs.

**Conclusions:**

No instrument has been developed and validated to assess the needs of informal caregivers of patients with cognitive impairment, whatever the stage and the etiology of the disease. As the perceived needs of caregivers may evolve with the progression of the disease and the dementia transition, their needs should be regularly assessed.

**Electronic supplementary material:**

The online version of this article (doi:10.1186/s12877-017-0481-9) contains supplementary material, which is available to authorized users.

## Background

The informal caregiver of people with cognitive impairment is often a spouse or a child, providing supervision, support and assistance with daily living activities during all stages of the disease to maintain the care recipient at home [1]. Prevalence of Mild Cognitive Impairment (MCI) ranges from 3 to 19% in adults older than 65 years [2]. In addition, the number of people with dementia in the world is expected to rise from 35.6 million in 2010 to an estimated 115.4 million in 2050 [3]. This will be associated with an increasing number of informal caregivers whose role represents a major societal and economic issue [4, 5].

Cognitive impairment, progressive loss of autonomy and behavioural disorders associated with the evolution of Alzheimer’s Disease and Related Diseases (ADRD) may lead to increased caregiver burden [6, 7]. This burden may have physical, psychological, emotional, social and financial impact on the informal caregiver [8]. Several studies have shown that caring for people with dementia was associated with depression, anxiety, greater risk of hypertension and heart disease, decreased immunity and higher mortality [9–13].

The increasing frailty of the caregiver has been shown to predict an early institutionalization of the patient over time [14]. Despite caregivers of people with dementia often providing intensive levels of assistance, their use of support services is low. A review showed that one third of caregivers did not use any service and one fourth caregivers used only one service [15]. In addition, previous studies have shown a large number of unmet needs were correlated with a higher burden and an increase in caregiver strain and depressive symptoms [16–23]. To ensure the service utilization by the caregivers and to minimize their burden, the supply (support, services) and the demand (caregivers’ needs) must be appropriate. Indeed, informal caregivers and professionals may differ in their perspectives to assess caregivers’ needs and in prioritising subsequent interventions and supports [24]. The assessment of the caregivers’ met and unmet needs represent a first step (i) to determine services or care plans for community-based programs and planning service delivery [25] (*policy purposes*); (ii) to refer caregivers to appropriate support and resources based on gaps-in needs identified and to ensure the service utilization (*clinical purposes*); and (iii) to design research programs for the caregivers (*research purposes*). This assessment can also be used when carrying out trials of interventions intended to improve caregiver outcomes including reducing unmet needs (*Research purposes*). Our research questions are: How to explore the caregivers’ needs of individuals with cognitive impairment and what methods are used?

A previous systematic review conducted in 2012, has provided an overview of the existing needs assessment instruments among people with cognitive impairment [26], but no such studies have been conducted considering the need assessment of their informal caregivers.

Our objective was to perform a systematic review of the methodologies used to identify the needs, the existing needs assessment instruments and the main topics of needs explored among caregivers of ADRD patients with mild cognitive impairment to dementia.

## Methods

### Search procedure/methods

Electronic databases and key articles were searched for studies published in English and French between January 1980 and January 2017. The searches were carried out in MEDLINE, PsycINFO, The Cochrane Library and Web of Science in order to identify quantitative and qualitative studies. We used the following search strategies for the research (detailed in Additional file 1): (carer*/ caregiver*/ loved one*/ famil*) AND (dementia/ Alzheimer*/ frontotemporal/ lewy/ vascular dementia/ cognitive impairment/ memory) AND (need*/ expectation*) AND (quantitative*/ qualitative*/ questionnaire*/ item*/ scale*/ tool*/ instrument*/ interview*/ cross-sectional/ focus group*/ structured/ verbatim* / survey). A manual search was performed at the end from the references of the included studies, from google scholar and using the ‘Related articles’ option on PubMed. The reference database used to retrieve records and for the screening was Endnote. This systematic review follows the *PRISMA statement* guidelines [27].

### Eligibility criteria

We included quantitative and qualitative studies that used questionnaires, instruments, focus group or semi-structured interviews to assess the needs (met and unmet needs) of informal caregivers. Informal caregivers were defined as unpaid, non-professionals who daily take care of individuals with mild cognitive impairment to dementia related to ADRD [1]. The included studies had to explore caregiver’s needs in terms of information, coping skills, support and services. We also included studies that used both quantitative and qualitative methods (mixed methods studies).

### Study selection

Two authors (T.N and C.M) independently screened the titles and abstracts of the citations identified by the search to determine which papers met the eligibility criteria. The final eligibility evaluation was performed utilizing the full paper. In cases with disagreements, discussions were held between authors until a consensus reached.

### Data collection

One review author (T.N) extracted the data using a data extraction form, and a second author (C.M) verified the data. The following data were extracted: year of publication, country in which the study was performed, first author, number and main characteristics of caregivers (proportion of females and spouses and the mean age), the care recipient health condition, the instrument aim, and the main characteristics of the needs assessment method.

## Results

### Selection of study

**Fig. 1:**
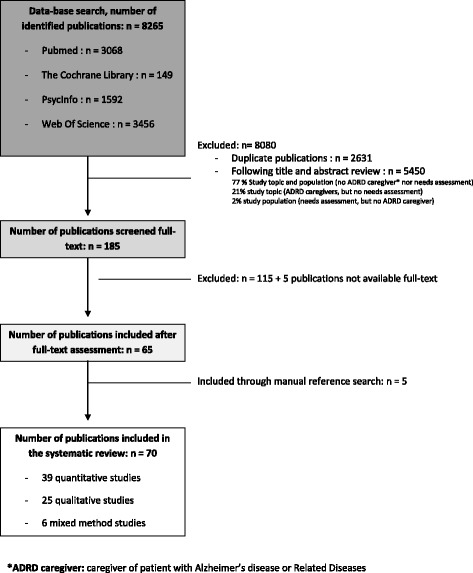
Systematic review flow-chart: selection of studies

A total of 8265 studies were identified through database searching. A flow-chart of the selection process is illustrated in Fig. 1. We excluded 8080 studies including 2631 duplicates and 5450 articles after screening of the title and abstract. The remaining 185 full text articles were screened independently and five additional studies were identified by the authors from the references of the included studies. A total of 70 studies were included.

### Study populations

A total of 11,122 informal caregivers was captured in the included studies: *n* = 9390 in quantitative studies, *n* = 1294 in qualitative studies, and *n* = 438 in mixed method studies (Tables 1, 2 and 3). In these studies, the caregiver needs assessment was performed according to the patient diagnosis in 19 studies, the stage of the disease in 45 studies or both in six studies. Nineteen studies were conducted among caregivers of Alzheimer’s Disease patients, four in Frontotemporal dementia, two in Lewy Body disease, one in vascular dementia and one in ADRD (diagnosis unspecified). The stage of the disease the most explored in the caregiver needs assessment was dementia: 38 studies with dementia caregivers and seven with early-onset dementia caregivers. Only four studies were conducted among MCI caregivers.

**Table 1:** Methodological characteristics of the included quantitative studies

Setting (Year / country)	Authors	CG sample	Needs assessment methods of the quantitative studies					
			Administration	Aim of the instrument	Domains - scale	Specific cognitive impairment	validated instrument	Recipient
1) In Mild Cognitive Impairment (MCI)								
MCI and AD								
*2010/USA* [21]	Ryan et al.	80 informal CGs (25 MCI and 55 AD); 68.8% female; 75% spouse; mean age = 64.1 years	Self-administered	Support and service needs	List of 18 services: medical, social and community, mental health, and other. Three-point rating scale.	yes	No, questionnaire developed for the study	CG
MCI and dementia								
2005/ Canada [37]	Aminzadeh et al.	141 informal CGs; 61.7% female; mean age = 60.1 years	Self-administered	CG’s needs, goals and outcomes	List of educational, support and care management needs (11 items).	yes	No, questionnaire developed for the study	CG
2011/USA [38]	Johnston et al.	15 CGS (8 dementia, 7 MCI)	By telephone	Patient’s and CG’s needs	15 care recipient need domains (77 items) and 4 CGs need domains (12 items): safety, management of cognitive and noncognitive symptoms, medical comorbidities, daily activities, CG education and support needs. Three-point rating scale.	yes	Yes - The Johns Hopkins Dementia Care Needs Assessment. Psychometric properties not formally tested / concurrent validity with QOL measures [39].	CG + patients
2) In Alzheimer’s Disease and Related Disorders (ADRD)								
2002/USA [40]	Wackerbarth et al.	128 informal CGs; 74.6% female; 34.7% spouse; mean age = 78.5 years	Self-administered	Information and support needs	Needs assessment surveys based on the results of 28 previously conducted in-depth interviews with CGs. Three sections: 20 information needs, 19 support needs and information about the caregiving experience. Four-point rating scale.	yes	No, questionnaire developed for the study	CG
AD								
1987/ USA [41]	Simonton	No participant	Self-administered	Information needs	15 questions with a five-point rating scale.	yes	No, questionnaire developed for the study	CG
1990/USA [42]	Fortinsky et al.	115 informal CGs (58 active CGs, 57 former CGs); 79.1% female; 47.8% spouse; mean age = 58 years	Self-administered	Information and service needs	Types of information and services desired and how much information was provided to them at the time of diagnosis.	yes	No, questionnaire developed for the study	CG
1992/USA [43]	Francis et al.	39 informal CGs; 72% female	By telephone	Service needs	2 parts: measures functional status on five dimensions (social resources, economic resources, mental health, physical health, and activities of daily living); measures 24 generically defined services received (intensity, provider) as well as perceived need.	No (elderly)	Yes - The Older American Resources and Services Multidimensional Functional Assessment Questionnaire (OMFAQ). Validity and reliability tested in elderly [44].	CG
1996/USA [45]	Bowd et al.	68 informal CGs (living in isolated communities); 73% female; 56% spouse; mean age = 64 years	Self-administered	Support needs	“Assessment of Perceived Needs of CGs»: 27 items (social and informal supports, the use of formal community services).	yes	No, questionnaire developed for the study	CG
1999/ Italy [19]	Dello Buono et al.	60 informal CGs; 50.6% female; 50% spouse; mean age = 76.83 years	Face-to-face interviews	service needs	A list of the local services (frequency of use, reason for non-use) and a list of ten interventions (choosing one or more which might be helpful).	No (elderly)	No, questionnaire developed for the study	CG
2006/USA [46]	Edelman et al.	100 informal CG living in rural communities; 61% female; 44% spouse; mean age = 61 years	Self-administered	information and service needs	A 22-item Checklist of Interest in Services and Information. Four areas: medical needs (6 items); care needs (5 items); coping needs (6 items); and service needs (3 items).	yes	No, questionnaire developed for the study	CG + patients
2007/ Finland [47]	Raivio et al.	1214 informal CGs; 63% female; 100% spouse; mean age = 78.2 years	Self-administered	service needs	11 areas: support and services received, the CGs’ subjective needs and satisfaction with these services.	No (elderly)	No, questionnaire developed for the study	CG
2011/ France [48]	Coudin et al.	74 informal CGs; 62% female; 74% spouse	Self-administered	difficulties, coping strategies and satisfaction	3 Questionnaires CADI-CAMI-CASI: the Carers Assessment of Difficulties index (30 items) - Managing Index (38 items) - Satisfaction Index (30 items).	No(CG)	Yes - CADI-CAMI-CASI Psychometric properties tested in CG of older people [49].	CG
2012/ France [50]	Amieva et al.	645 informal CGs; 61.1% spouse	Self-administered	CG’s needs	28-item questionnaire. Four main needs: learning skills to improve daily life management of their relatives (7 items); information on the disease (7 items); improving CGs’ self-confidence (7 items); improving communication (7 items).	No (elderly)	Yes - Echelle d’attentes en matière de consultations (EAC) Psychometric properties tested in CGs of depressed elderly people (Cronbach’s alpha 0.89 to 0.95, intra-class coefficient 0.92) [51].	CG
Frontotemporal Dementia (FTD)								
2011/ Canada [52]	Chow et al.	79 informal CGs; 57% female; median age = 58 years	Web-based survey	CG’s needs	9 questions with multiple choice responses.Areas: diagnosis, symptoms, the troublesome aspects of caregiving, resources/interventions, process of learning about FTD.	yes	No, questionnaire developed for the study	CG
2013/ Germany [53]	Diehl-Schmid et al.	94 informal CGs; 72% female; 80% spouse; mean age = 89.11 years	Self-administered	Support and service and intervention needs	List of 45 support services and interventions relating to the following domains: information, psychosocial support for relatives, care outside of home, support at home, financial support, safety issues, therapies for the patients, and raising awareness. CGs were asked to rate the helpfulness of the proposed services and interventions.	yes	No, instrument developed for the study	CG
AD and FTD								
2010 / Australia [54]	Nicolaou et al.	30 FTD dyads; 93% female94% spouse; mean age = 58.5 years	Face-to-face interviews	Patient’s and CG’s needs	24 areas and four categories: autonomy; physical needs; psychological, emotional and social needs; and CG’s needs.	No (elderly)	Yes - Camberwell Assessment of Need for the Elderly (CANE) Psychometric properties tested in PWD and CGs of PWD [55].	CG
Lewy Body Dementia								
2011 / USA [56]	Galvin et al.	962 CGs; 87,9% female; 40,6% spouse; mean age = 55.9 years	Web-based survey	Functional, behavioral and affective disturbances burden	Areas: ADL, IADL, paid services used and requested services by CGs.	yes	No, questionnaire developed for the study	CG
2015/UK [57]	Killen et al.	122 CGs; 89% female; 17.6% spouse	Web-based survey	Information and support needs	Ten short questions focused on past support and information experiences, difficulties encountered that could benefit from information and support, and appropriate topics for inclusion in future resource development.	yes	No, questionnaire developed for the study	CG + patients
AD and vascular dementia								
2001/ USA [58]	England	92 filial CGs; 72.8% female; mean age = 53.45 years	Face-to-face interviews	Learning and resource needs	Checklist of learning and resource needs: prioritization of a list of 15 information requests and prioritization a list of ten resource requests.	yes	No, questionnaire developed for the study	CG
3) Early-onset Dementia (EOD)								
2012/ Norway [22]	Rosness et al.	45 informal CGs68.9% female	Self-administered	Burden and needs	A 20-item questionnaire (Care-EOD) assessing the burden and needs. Six-point rating scale.	yes	No, questionnaire developed for the study	CG
2014/ USA [59]	Gibson et al.	81 CG; 76.5% female; 69.2% spouse	Web-based survey	Service and support needs	Areas: caregiving obligations, utilization of services, perceived importance of services, employment status, need for and access to financial services and benefits, and perceived understanding of the experiences of CGs by the public and by service providers.	yes	No, survey developed for the study	CG
4) Dementia								
1995/ UK [60]	Philp et al.	114 informal CGs; 86% female; 22.8% spouse; mean age = 80.7 years	Face-to-face interviews	Service needs	List of locally available services: frequency of use, number of hours of support a week.	yes	No, instrument developed for the study	CG
1999/ UK [61]	Turner et al.	30 CGs; 60% female; 37% spouse; 2/3 < 65 years	Face-to-face interviews	Training needs	Four areas (19 items): practical advice, information, coping with caring, coping with the person with dementia.	yes	No, survey developed for the study	CG
2004/USA [62]	Gaugler et al.	694 informal CGs (344 community PWD, 144 institutional PWD, 216 deceased PWD); 70.9% female;37.8% spouse; mean age = 61.17 years	Self-administered	Unmet needs	7 items, 34 questions: help with ADL tasks, help with IADL tasks, dementia symptoms, timing of care, formal support, information and confidante/family support. Yes/no questions.	yes	No, questionnaire developed for the study	CG
2005/ Austria [16]	Wancata et al.	45 informal CGs73% female46% spousemean age = 60.9 years	Interview	CG’s needs	18 problem areas with several possible interventions (+ an optional area for additional problems). Five-point rating scale.	yes	Yes - Carers’ Needs Assessment for Dementia (CNA-D) Psychometric properties tested in CGs of PWD [16].	CG
2008/ UK [63]	Orrell et al.	81 informal CGs	Face-to-face interviews	Patient’s and CG’s needs	24 areas and four categories: autonomy; physical needs; psychological, emotional and social needs; and CG’s needs.	No (elderly)	Yes - Camberwell Assessment of Need for the Elderly (CANE) Psychometric properties tested in PWD and CGs of PWD [55].	CG + patients + professionals
2008 / 2009/ Netherlands [55, 18]	Van der Roest et al.(2 studies)	322 informal CGs; 68.6% female; 54.3% spouse; mean age = 65.4 years	Face-to-face interviews	Patient’s and CG’s needs	24 areas and four categories: autonomy; physical needs; psychological, emotional and social needs; and CG’s needs.	No (elderly)	Yes - Camberwell Assessment of Need for the Elderly (CANE) Psychometric properties tested in PWD and CGs of PWD [55].	CG + patients
2009/ UK [64]	Selwood et al.	113 informal CGs; 67.9% female; 33.9% spouse; mean age = 61.2	Self-administered	Strategies to reduce abusive behavior	List of 14 strategies which had either helped them already or that they thought would have the potential to help them avoid the abusive behaviors. Rating of the most helpful and the second most helpful strategies.	yes	No, instrument developed for the study	CG
2008/ USA [65]	Nichols et al.	165 informal CGs; 78.4% female; 50.3% spouse; mean age = 64.7 years	Selection of a topic to discuss by CG	Behavior, stress and coping pressing concerns	25 pamphlets addressing particular behaviors and 12 pamphlets on CG stress/coping and improving the CG’s own well-being.	yes	No, instrument developed for the study	CG
2010/ Netherlands [66]	Peeters et al.	984 informal CGs; 71.6% female; 50.8% spouse; mean age = 62.8 years	Self-administered	Professional support needs	2 parts: 14 areas (30 items) on problems that are faced by persons with dementia and problems experienced by CGs; 56 items about the needs for additional professional support.	yes	No, questionnaire developed for the study	CG
2010/Italy [17]	Rosa et al.	112 informal CGs; 69% female; mean age = 55 years.	Self-administered	CG’s needs	“Questionnaire assessing the CGs’ needs”. Four areas (22 items): need of medical relevance, educational needs, need of emotional and psychological support, need of services.	yes	No, questionnaire developed for the study	CG
2011 and 2016/ USA [24, 67]	Koenig et al. Steiner et al.	33 informal CGs; 29 female; 11 spouse; mean age = 62.2 years	Face-to-face interviews	information needs	List of 48 items: 25 items related to needing help for the care recipient +23 items related to needs of the CG. Participants were asked to choose their top ten needs.	yes	No, instrument developed for the study	CG
2012/ USA [68]	Li	208 informal CGs; 57.7% female; mean age = 49.7 years	By telephone	unmet service needs	14-service item survey about self-care, coping, decision making, CG training and others. Yes/no questions.	No (CGs)	No, instrument developed for the study	CG
2013/USA [20]	Black et al.	246 informal CGs; 74,8% female; 41,5% spouse; mean age = 66,1 years	Face-to-face interviews	Patient’s and CG’s needs	15 care recipient need domains (77 items) and 4 CGs need domains (12 items): safety, management of cognitive and noncognitive symptoms, medical comorbidities, daily activities, CG education and support needs. Three-point rating scale.	yes	Yes -The Johns Hopkins Dementia Care Needs Assessment. Psychometric properties not formally tested / concurrent validity with QOL measures [39].	CG + patients
2013/ UK [69]	Miranda-Castillo et al.	128 informal CGs; 71.1% female; 64.1% spouse	Face-to-face interviews	Patient’s and CG’s needs	24 areas and four categories: autonomy; physical needs; psychological, emotional and social needs; and CG’s needs.	No (elderly)	Yes - Camberwell Assessment of Need for the Elderly (CANE) Psychometric properties tested in PWD and CGs of PWD [55].	CG + patients + professionals
2013/ Netherlands [70]	Zwaanswijk et al.	1494 informal CGs (caregiving <1 year =89, 1-4 years =744 and >4 years =661); 71.7% female; 58.2% spouse.	Self-administered	Professional support needs	35 problems and 59 needs for additional support (currently received and needs).	yes	No, questionnaire developed for the study	CG
2015/ USA [23]	Jennings et al.	307 informal CGs; 64% female; 41% spouse	Self-administered	Care needs and self-efficacy	Nine-item scale with three domains: perception of the primary care provider, advice on dementia-related topics and self-efficacy for caring and for accessing help. Five-point rating scale.	yes	No, scale developed for the study	CG + professionals
2015/ USA [30]	Sadak et al.	Baseline: 130 CGs; 80% female; 63% spouse; mean age = 66 years. Re-test: 79 CGs	Self-administered	Knowledge and skills	35 items (23 “knowledge” and 12 “skills” items). 5-level Likert type response scale.	yes	Yes - Partnering for Better Health - Living with Chronic Illness: Dementia (PBH-LCI:D) Psychometric properties tested in CGs of PWD (Cronbach’s alpha 0.95) [30].	CG

*AD* Alzheimer’s Disease, *ADRD* Alzheimer’s Disease and Related Disorders, *CG* caregiver, *EOD* Early-Onset Dementia, *FTD* FrontoTemporal Dementia, *MCI* Mild Cognitive Impairment, *PWD* People With Dementia, *QOL* Quality Of Life

**Table 2:** Methodological characteristics of the included qualitative studies

Setting (Year / country)	Authors	CG sample	Needs assessment methods of the qualitative studies
1) In Alzheimer’s Disease and Related Disorders (ADRD)			
AD			
1997/ USA [71]	Beisecker et al.	104 informal CGs; 70.2% female61.5 spouse; mean age = 63.6 years	Semi-structured interview by telephone. Topics: physician-patient-CG interactions, informational needs and advance directives.
1999/ USA [72]	Loukissa et al.	34 CGs; 74% female 42% spouse; 68% african-american CGs	5 focus groups. The open-ended interview began with: “If you were to write a book for persons in similar situations to yours, what would you want them to know?”.
2001/ USA [73]	Smith et al.	45 informal CGs; 87% female51% spouse	Semi-structured intensive interview. Nine questions about needs, changes, experiences, skills and assistance.
2003/ USA [74]	Farran et al.	177 informal CGs	Semi-structured interviews and open-ended group discussion (during group session). Topics: care recipient issues and concerns expressed by CGs; specific skills needed by CGs to address key care recipient issues.
2004/ USA [75]	Farran et al.	177 informal CGs	Semi-structured interviews and open-ended group discussion (during group session). Topics: CG issues and concerns discussed by CGs; specific skills needed by CGs to address key CG issues and concerns.
FTD			
2013/ Canada [76]	Nichols et al.	14 young CGs (ten female, aged 11-18).	2 focus groups using a semi-structured interview guide. Topics: experiences and needs of young CG at various points in the patient’s diagnostic process and course of illness (to create a relevant support website).
2) Early-onset Dementia (EOD)			
2010/ Netherlands [77]	Bakker et al.	1 informal CG; female; spouse; 46 years	A single case study design (qualitative interviews with the CG). Topics: experiences and needs during period prior to diagnosis, diagnosis, period after diagnosis, caring, transitions in care and future perspective.
2014/ The Netherlands [78]	Millenaar et al.	14 children CGs; eight female; mean age = 21.0 years	Semi-structured interviews. Topics: the children’s reactions to the diagnosis, the help they received after the diagnosis, and the resulting changes in their lives. Topics focused on the children’s needs.
2015/ Netherlands [79]	Boots et al.	28 informal CGs; 75% female; 78% spouse; mean age = 63.6 years	4 focus group interviews using context-mapping approach. Topics: needs and wishes to prevent overburdening, need for care and need for communication of care.
2017/Canada [80]	Wawrziczny et al.	40 spouses of persons with EOD and 38 spouses of persons with late-onset dementia; 23 and 20 female; mean age = 57.4 and 77.0	Semi-structured interviews based on the French version of the Carers Outcome Agreement Tool. Four areas: the types of information and support provided, the changes that could improve the quality of life for the PWD and the spouse CG, the quality of the aid received, and the desire for future assistance.
3) Dementia			
1986/ Sweden [81]	Brâne	56 informal CGs (28 with patient in early phase and 28 with patient in long stay wards)	Interviews and two group meeting.Topics: situation, need of help, feelings about the care on the long stay ward.
2001/ USA [82]	Lampley-Dallas et al.	13 informal CGs (african-american CGs); 11 female; two spouse; mean age = 54 years	2 focus group. 3 questions about needs, the health care system (help and interaction) and stress.
2003/ India [83]	Shaji et al.	17 informal CGs; 76% female	Semi-structured interviews. Topics: demographic data, level of knowledge about AD, the practical and psychological problems of CGs and their attitudes towards caring.
2005/ UK [84]	Innes et al.	30 informal CGs (rural CGs); 22 female	16 semi-structured interviews and three focus group. Topics: services used, perceived benefits and drawbacks of each service, alternative sources support, views on the impact of geographical location and service use and support.
2009/ Netherlands [85]	de Jong et al.	Nine informal CGs; five female; four spouse	Semi-structured interviews in their home or by telephone. Topics: needs and wishes of CGs using a skilled psychogeriatric day-care facility; functioning of the CGs and the PWD, health care, knowledge about dementia and CGs’ experience.
2011/ Australia [86]	Shanley et al.	15 CGs; eight female; ten spouse; mean age = 64 years	Semi-structured interviews. Topics: history of caregiving experience; ‘quality of life’ and ‘quality of care’; particular challenges encountered; sources of advice and support; the needs of CGs; and the positive and negative aspects of formal service provision.
2012/ USA [87]	Samia et al.	Survey: 168 informal CGs; 84.5% female; 45.2% spouse; mean age = 66,6 yearsFocus group: 26 family CGs; 84.6% female; 60% spouse	A multi-stage qualitative descriptive study: open-ended survey and five focus group. Topics: ongoing training needs and preferences of previously trained CGs.
2013/ Australia [88]	Low and White et al.	31 CGs; 27 female; 21 spouse; mean age = 63 years	Face-to-face, telephone or group interviews. Topics: characteristics of an ideal dementia-specific community care service, the ideal outcomes or achievements of a dementia-specific community care service.
2013/ Singapor [89]	Vaingankar et al.	63 informal CGs; 60% female20% spouse; mean age = 52.9 years	Ten focus group and 12 semi-structured interviews (funnel approach). Topics: experiences and discussion focused on each identified unmet needs or challenges.
2014/ Germany [90]	Muders et al.	85 CGs	Questionnaire with two open-ended questions. Topics: exploration and documentation of the CG’s needs and identification of the healthcare professionals to adequately support them.
2015/ USA [91]	Meyer et al.	Ten vietnamese informal CGs; seven female; two spouse; mean age = 55	Semi-structured interviews (*n* = 10 CGs). Topics: family structure and immigration, beliefs about dementia, experiences with caregiving, coping strategies, help-seeking and service utilization.1 Focus group (*n* = 5 CGs). Topics: helpful interventions or other treatments to reduce CG distress, sources of stress and coping/management strategies.
2016/Thailand [92]	Griffiths et al.	30 CGs; 24 female; 12 spouse	Semi-structured interviews. Topics: problems and needs of CGs who help older people with dementia to do activities of daily living.
2016/USA [93]	Peterson et al.	27 CGs; 19 female; eight spouse; mean age = 58.5	Semi-structured interviews. Topics: caregiving characteristics, care recipient symptoms, information regarding diagnosis, care issue and strategies (trigger, previous sources, most helpful sources, barriers, expectations and preferences), preferred learning methods and setting, and the use of technology.
2016/USA [94]	Samson et al.	32 African American CGs; 28 female	4 focus group. Topics: examination of the concerns and experiences of the African American CGs (differences with other racial or ethnic groups) + identification of the information needs and preferences for information, education and support.
2016/USA [95]	Jennings et al.	36 CGs; 26 female; 24 spouse; mean age = 63	4 Focus group with CGs. Topics: goals in dementia care relating to specific domains, including medical care, social functioning, safety, and end-of-life care.

*AD* Alzheimer’s Disease, *ADRD* Alzheimer’s Disease and Related Disorders, *CG* caregiver, *EOD* Early-Onset Dementia, *FTD* FrontoTemporal Dementia, *MCI* Mild Cognitive Impairment, *PWD* People With Dementia

**Table 3:** Methodological characteristics of the included mixed method studies

Setting (Year / country)	Authors	CG sample	Needs assessment methods of the mixed method studies					
			Administration	Aim of instrument	Domains	Specific cognitive impairment	Validated instrument	Recipient
Cognitive impairment (MCI, dementia and other)								
2010/ Netherlands [96]	Wolfs et al.	252 informal CGs; 62.7% female; 45.2% spouse; mean age = 61.9 years	By telephone	care needs and satisfaction	Semi-structured interviews by telephone including quantitative and qualitative data. Eight areas: informal caregiving and burden; inventory of the utilized care and treatment and satisfaction; inventory of the non-utilized reasons for non-utilizing care and treatment option; needs to improve care and treatment; the choice and the transparency of the care and treatment option.	yes	No, instrument developed for the study	CG
AD								
1998/ USA [97]	Kuhn et al.	20 informal CGs; 11 female 14 spouse; mean age = 60 years	Self-administered	topic of interest	16-item survey (learning needs in relation to the disease). Three-point rating scale. + Semi-structured interviews. Seven areas, 20 open-ended questions: symptoms of AD and initial perception, CG reaction to diagnosis and beliefs about AD, needs, changes, coping, use of formal and informal resources, future planning and advice.	yes	No, instrument developed for the study	CG
2005/ USA [98]	Habermann et al.	20 informal CGs; 16 female; 12 spouse; mean age 60.9 years	Face-to-face interviews	CG’s needs	16-item survey with three areas (Caregiver Assistance Measure): caregiving knowledge and skills, community resources, self-care. Three-point rating scale. + Open-ended questions: most difficult aspects of the caregiving situation, type of assistance perceived as important.	yes	No, instrument developed for the study	CG
EOD								
2014/ Canada [99]	Ducharme et al.	32 informal CGs; 75% female78% spouse; mean age 54,28 years	Face-to-face interviews	unmet support needs	Questionnaire FCSA (4 areas, 38 items) + semi-structured interview. Topics: identification of other needs not covered by the tools and other types of useful help, improvement and quality expectation of help.	No(CG)	Yes - Family CGs Support Agreement (FCSA) tool	CG
Dementia								
2001/ Australia [100]	Leong et al.	Survey: 94 CGs; over 2/3 were female; mean age = 65.5 yearsQualitative interviews: 10 CGs; five female	Self-administered	CG’s needs	Questionnaire FCNS. Eight areas, 42 items: information, household, spiritual, Respite, personal, psychological, legal and financial, and physical care/ skills needs. + Semi-structured interviews. Topics: the nature of family caring and gather a more complete picture of experiences, feelings, perceptions and needs of CGs)	yes	No, Family Carer Need Survey (FCNS).This instrument draws on the Home Carer Need Survey, with modifications to suit Australian respondents caring for a family member with dementia.	CG
2010/ Australia [101]	Stirling et al.	20 informal CGs; 18 female 14 spouse	By telephone	services needs	Community service use measured explored by the final domain of the Carer’s checklist + 3 semi-structured interviews. Topics: CG interaction with community service providers, CG experiences, CG socio-economic circumstances, felt needs for services.	No(CG)	Yes - The Carer’s checklist	CG

*AD* Alzheimer’s Disease, *CG* caregiver, *EOD*, Early-Onset Dementia, *MCI* Mild Cognitive Impairment

### Methodologies to identify the needs of informal caregivers

The sample of selected studies was composed of 39 quantitative studies, 25 qualitative studies and six mixed method studies. Tables 1, 2 and 3 summarize the methodology characteristics of the studies (quantitative, qualitative and mixed research).

#### Quantitative studies

A majority of the needs assessment instruments were especially developed for the research (28/39 studies) (Table 1). Eleven studies have used validated instruments developed for the research or clinical use to assess the needs of elderly caregivers (6/11), dementia patient and caregivers (2/11), dementia caregivers (2/11) and nonspecific caregivers (1/11). Some of the validated instruments developed to assess the needs of patients with dementia, such as the Camberwell Assessment of Needs for the Elderly (CANE) [28], The Johns Hopkins Dementia Care Needs Assessment (JHDCNA) by Black et al. and the Care needs assessment pack for dementia (CarenapD) [29], include several questions concerning the caregivers’ needs. Only one valid and reliable instrument was identified to specifically assess the dementia caregivers’ needs: the Carers’ Needs Assessment for Dementia (CNA-D) [16]. They used focus groups and in-depth interviews with both caregivers and experts, along with a literature search, to design this assessment tool. This semi-structured research interview included 18 problem areas. For each problem area, several possible interventions are proposed: individual psychoeducation, psychoeducational group, self-help group for family members, printed information material, and other intervention. A second instrument called Partnering for Better Health - Living with Chronic Illness: Dementia (PBH-LCI:D) was used to evaluate the acquired knowledge and skills allowing to indirectly assess the dementia caregiver’s needs [30]. This instrument with 35 items was not specifically developed to needs assessment. It explored the acquired knowledge and skills allowing to indirectly assess the dementia caregiver’s needs.

#### Qualitative studies

Several methodologies were used to identify the needs of informal caregivers: semi-structured interviews (10/25 studies), focus group (6/25 studies), survey with open-ended questions (1/25 studies), case study (1/25) and mixed qualitative methods as semi-structured interviews/focus group (6/25 studies) or open-ended survey/focus group (1/25 studies) (Table 2). The main objectives of these qualitative studies were to explore the experiences of caregivers with caring and community services, their information, training and support needs, their satisfaction with services and the gap between their perceived needs and the proposed services.

#### Mixed method studies

Six studies including quantitative and qualitative methodologies were conducted among informal caregivers (Table 3). The majority of the studies consisted of quantitative survey associated with a semi-structured interview to explore the caregiver’s experience and needs or to complete and to comment the quantitative data. The quantitative instruments were either developed for the study, or derived from validated instrument for nonspecific caregivers.

### Main topics of needs explored in the quantitative and mixed method studies

From the 45 studies using quantitative methods (39 quantitative and six mixed methods research), 36 instruments were described (wholly or in part), four studies used the same validated instruments (CANE or JHDCNA) and four were not described. Table 4 summarizes the main items of needs explored among the different instruments. Twenty-seven items were related to five areas of needs: information (e.g. on the disease and the treatment) psychological (e.g. emotional support for caregivers and their relative), social (e.g. financial issue and community services), psychoeducational (e.g. coping skills and caregiver training) and other needs (e.g. medication management and environment safety). The most explored topics of needs (≥50%) in caregiver needs assessment instruments were: information on the disease (78%), support for the caregivers (64%), coping for caring (56%), community services related to patient care (50%), financial issue (50%) and safety/supervision (50%).

**Table 4:** Main topics of needs explored in instruments of the included studies

Items	No. of instruments who reported the item -n (%) *n* = 36 instruments	References of the instruments who reported each item
Information needs		
On the disease (e.g., cognition, behavioural disorders, dementia)	28 (78)	[16, 20, 22, 23, 30, 37, 38, 40–42, 45, 46, 48, 50, 52–55, 58, 62, 64, 66, 67, 69, 70, 97–99]
On the pharmacological treatment	17 (47)	[16, 17, 22, 30, 40–42, 46, 50, 56, 64, 65, 67, 96–99]
On the non-pharmacological treatment	3 (8)	[47, 53, 96]
On the available services	10 (28)	[16, 23, 37, 40, 61, 64, 66, 70, 98, 99]
Psychological needs		
Support for the caregiver (psychological and emotional support)	23 (64)	[16, 17, 20, 22, 38, 40, 42, 43, 45, 46, 48, 52–56, 60, 64–67, 69, 70, 96, 98]
Support for the patient (psychological and emotional support)	8 (22)	[19, 40, 57, 58, 60, 66, 70, 96]
Social interactions, company	11 (31)	[16, 37, 40, 43, 45, 48, 58, 62, 65, 70, 99]
Time for themselves	9 (25)	[16, 45, 48, 56, 65, 67, 68, 98, 99]
Social needs		
Institutionalization	9 (25)	[22, 46, 53, 58, 64, 66, 68, 70, 96]
Financial issue	18 (50)	[16, 19, 22, 40–42, 46–48, 52, 53, 56, 62, 64, 67, 97, 98]
Legal issue	14 (39)	[16, 30, 40–43, 46, 52, 53, 56, 62, 66, 70, 97]
Respite, Day care	14 (39)	[19, 22, 41, 42, 45, 47, 52, 53, 56, 58, 60, 64, 65, 70]
Community services:	8 (22)	[20, 38, 41, 42, 46, 48, 61, 96, 97]
related to home support (meal, housework, transport)	15 (42)	[16, 23, 43, 45, 47, 53, 56, 58, 60, 62, 64, 66, 68, 70, 98]
related to patient care	18 (50)	[19, 23, 37, 40, 41, 43, 45, 47, 53, 56, 58, 60, 62, 64, 66, 69, 70, 98]
Psycho educational needs		
Coping with behavioural disorders	16 (44)	[16, 17, 23, 41–43, 46, 56, 61, 65–68, 70]
Coping with cognitive disorders	13 (36)	[17, 23, 30, 46, 56, 61, 62, 64, 67, 70]
Coping with patient feelings	8 (22)	[30, 40, 56, 62, 65–67, 70]
Coping for caring	20 (56)	[16, 17, 22, 37, 40, 45, 46–48, 52, 53, 56, 62, 64, 67, 97, 99]
Communication with patient	12 (33)	[16, 17, 24, 37, 40, 46, 50, 57, 61, 65, 67, 70, 97]
Stimulating/appropriate activities	13 (36)	[41, 42, 46, 48, 50, 57, 65, 66, 68, 70, 96, 97, 99]
Caregiver training	16 (44)	[16, 22, 30, 37, 42, 43, 50, 52, 53, 56, 58, 62, 65, 67, 98, 99]
Other needs		
Environmental Safety (material, device), supervision	18 (50)	[19, 22, 23, 41, 43, 47, 48, 53, 58, 60–62, 65–67, 70, 98, 99]
Incontinence	6 (17)	[42, 48, 61, 65, 67, 68]
Caregiver general health	8 (22)	[16, 20, 22, 24, 37, 38, 48, 56, 65, 67]
Medication management	7 (19)	[30, 40, 56, 57, 62, 67, 98]
Sexuality/Intimacy	5 (14)	[46, 56, 62, 65, 99]

## Discussion

This article provides the first overview of existing needs assessments methods in caregivers of individuals with ADRD developed for clinical or research use. Despite the large number of studies include in the analysis, only one instrument was validated to assess the needs of dementia caregivers: the Carers’ Needs Assessment for Dementia (CNA-D) [16]. No validated instrument was found for the caregivers of individuals with ADRD in the others stages of the disease progression. Moreover, no quantitative nor qualitative study has assessed the needs of caregivers of individuals with preclinical symptoms of ADRD. The caregivers’ needs identification was often performed in the dementia area in comparison with the MCI area (39 studies versus four studies). Similarly, few studies were conducted among frontotemporal dementia, vascular dementia and Lewy Body disease caregivers (6 studies) compared to Alzheimer’s disease caregivers (19 studies).

Many quantitative instruments were used in research area to develop programs and interventions tailored to the caregivers’ needs or in clinical area to identify their needs and to offer them appropriate support. Caregivers’ needs for services or care plans and referring to support were included in many developed instruments. The items of corresponding needs were as follows: psychological and emotional support (64%), information about financial and legal issues (50 and 39%), information about respite and day care (39%) and community services related to patient care (50%) and home support (42%). Despite the diversity of the items present in the identified instruments, they allow to explore a larger number of topics such as information, skills, support and service needs, compared with qualitative methods. However, qualitative research produces large amounts of textual data in the form of transcripts and observational field notes about a predetermined topic [31]. Qualitative research methods have used in the social sciences and deserve to be an essential component in health and health services research. They allow exploring individually or in groups the perceived needs of caregivers. Unlike quantitative methods, qualitative methods allow to explore in depth specific needs (e.g. needs in an ethnic population, needs of interactions with physician and care providers, changing needs before, during and after diagnosis) and experiences of caregivers (e.g. experiences with support and services). Understanding these challenges may lead to improve the health care provision for informal caregivers. Thus, qualitative and quantitative approaches are complementary: qualitative work may be conducted as a preliminary to quantitative research, used to supplement quantitative work or used to explore complex phenomena or areas not amenable to quantitative research [32].

The caregiver plays a crucial role across all stages of the progression and identifying their needs should be performed by the health professionals at each stage to prevent or reduce their burden. Many studies have assessed the effectiveness of interventions on caregiver burden and psychological disorders [33–36]. The meta-analysis of Pinquart et al. including 127 interventions showed a significant but small effects on burden, depression, subjective well-being, and knowledge and/or coping abilities of the caregiver [34]. There is a lack of systematic investigations of the efficacy of treatment combinations using a needs assessment in caregivers of individuals with subjective cognitive impairment, MCI or dementia. To our knowledge no study has shown that caregivers receive the interventions matching their needs assessed as outcome with a validated instrument.

This current review has some limitations. The main restrictions concerned the searching process. Only studies published in scientific journals were included in the systematic review. The needs assessments of caregivers of individuals with cognitive impairment published in the “grey literature” were not explored. The searching process was also limited to a number of databases which covered both the clinical and psychosocial aspects of the study. Another limitation in the analysis of the different needs assessment instruments since the topic of explored needs were not always fully described in the included studies.

## Conclusion

To reduce the caregiver burden and to facilitate the patient’s home care, policymakers, service planners, health professionals and researchers must understand the needs of this growing population. This systematic review highlights the necessity to develop a validated instrument to assess the met and unmet needs of informal caregivers of patients with a cognitive impairment across all stages of the disease progression and whatever the etiology. As the perceived needs of caregivers may evolve with the progression of the disease and the dementia transition, the needs should be regularly assessed and taking into account the needs for information, coping skills, support and service.

## Additional file

Additional file 1: Search strategies. This file provide the search strategies used in MEDLINE, PsycINFO, The Cochrane Library and Web of Science in order to identify quantitative and qualitative studies for the systematic review. (DOC 25 kb)

## References

[1] Brodaty H, Donkin M (2009). Family caregivers of people with dementia. Dialogues Clin Neurosci.

[2] Gauthier S, Reisberg B, Zaudig M, Petersen RC, Ritchie K, Broich K (2006). Mild cognitive impairment. Lancet Lond Engl.

[3] Alzheimer’s Disease International. The World Alzheimer Report 2010: The Global Economic Impact of Dementia. [Internet]. London; 2010. Disponible sur: http://www.alz.org/documents/national/world_alzheimer_report_2010.pdf. Accessed 15 June 2016.

[4] Joling KJ, Schöpe J, van Hout HPJ, van Marwijk HWJ, van der Horst HE, Bosmans JE (2015). Predictors of Societal Costs in Dementia Patients and Their Informal Caregivers: A Two-Year Prospective Cohort Study. Am J Geriatr Psychiatry.

[5] König H-H, Leicht H, Brettschneider C, Bachmann C, Bickel H, Fuchs A (2014). The costs of dementia from the societal perspective: is care provided in the community really cheaper than nursing home care?. J Am Med Dir Assoc.

[6] Dauphinot V, Delphin-Combe F, Mouchoux C, Dorey A, Bathsavanis A, Makaroff Z (2015). Risk factors of caregiver burden among patients with Alzheimer’s disease or related disorders: a cross-sectional study. J Alzheimers Dis.

[7] Dauphinot V, Ravier A, Novais T, Delphin-Combe F, Moutet C, Xie J (2016). Relationship Between Comorbidities in Patients With Cognitive Complaint and Caregiver Burden: A Cross-Sectional Study. J Am Med Dir Assoc.

[8] Zarit SH, Reever KE, Bach-Peterson J (1980). Relatives of the impaired elderly: correlates of feelings of burden. The Gerontologist.

[9] Schulz R, O’Brien AT, Bookwala J, Fleissner K (1995). Psychiatric and physical morbidity effects of dementia caregiving: prevalence, correlates, and causes. The Gerontologist.

[10] Cooper C, Balamurali TBS, Livingston G (2007). A systematic review of the prevalence and covariates of anxiety in caregivers of people with dementia. Int Psychogeriatr.

[11] von Känel R, Mausbach BT, Patterson TL, Dimsdale JE, Aschbacher K, Mills PJ (2008). Increased Framingham Coronary Heart Disease Risk Score in dementia caregivers relative to non-caregiving controls. Gerontology.

[12] Kiecolt-Glaser JK, Dura JR, Speicher CE, Trask OJ, Glaser R (1991). Spousal caregivers of dementia victims: longitudinal changes in immunity and health. Psychosom Med.

[13] Schulz R, Beach SR (1999). Caregiving as a risk factor for mortality: the Caregiver Health Effects Study. JAMA.

[14] Yaffe K, Fox P, Newcomer R, Sands L, Lindquist K, Dane K (2002). Patient and caregiver characteristics and nursing home placement in patients with dementia. JAMA.

[15] Brodaty H, Thomson C, Thompson C, Fine M (2005). Why caregivers of people with dementia and memory loss don’t use services. Int J Geriatr Psychiatry.

[16] Wancata J, Krautgartner M, Berner J, Alexandrowicz R, Unger A, Kaiser G (2005). The Carers’ Needs Assessment for Dementia (CNA-D): development, validity and reliability. Int Psychogeriatr.

[17] Rosa E, Lussignoli G, Sabbatini F, Chiappa A, Di Cesare S, Lamanna L (2010). Needs of caregivers of the patients with dementia. Arch Gerontol Geriatr.

[18] van der Roest HG, Meiland FJM, Comijs HC, Derksen E, Jansen APD, van Hout HPJ (2009). What do community-dwelling people with dementia need? A survey of those who are known to care and welfare services. Int Psychogeriatr.

[19] Dello Buono M, Busato R, Mazzetto M, Paccagnella B, Aleotti F, Zanetti O (1999). Community care for patients with Alzheimer’s disease and non-demented elderly people: use and satisfaction with services and unmet needs in family caregivers. Int J Geriatr Psychiatry.

[20] Black BS, Johnston D, Rabins PV, Morrison A, Lyketsos C, Samus QM (2013). Unmet needs of community-residing persons with dementia and their informal caregivers: findings from the maximizing independence at home study. J Am Geriatr Soc.

[21] Ryan KA, Weldon A, Huby NM, Persad C, Bhaumik AK, Heidebrink JL (2010). Caregiver support service needs for patients with mild cognitive impairment and Alzheimer disease. Alzheimer Dis Assoc Disord.

[22] Rosness TA, Haugen PK, Gausdal M, Gjøra L, Engedal K (2012). Carers of patients with early-onset dementia, their burden and needs: a pilot study using a new questionnaire--care-EOD. Int J Geriatr Psychiatry.

[23] Jennings LA, Reuben DB, Evertson LC, Serrano KS, Ercoli L, Grill J (2015). Unmet needs of caregivers of individuals referred to a dementia care program. J Am Geriatr Soc.

[24] Steiner V, Pierce LL, Salvador D (2016). Information Needs of Family Caregivers of People With Dementia. Rehabil Nurs J.

[25] Eifert E, Eddy J (2012). The role of needs assessments in enhancing support service utilization by family caregivers of persons with Alzheimer’s disease. Am J Health Stud.

[26] Schmid R, Eschen A, Rüegger-Frey B, Martin M (2012). Instruments for comprehensive needs assessment in individuals with cognitive complaints, mild cognitive impairment or dementia: a systematic review. Int J Geriatr Psychiatry..

[27] Moher D, Liberati A, Tetzlaff J, Altman DG, PRISMA Group (2009). Preferred reporting items for systematic reviews and meta-analyses: the PRISMA statement. J Clin Epidemiol.

[28] Reynolds T, Thornicroft G, Abas M, Woods B, Hoe J, Leese M (2000). Camberwell Assessment of Need for the Elderly (CANE). Development, validity and reliability. Br J Psychiatry J Ment Sci.

[29] McWalter G, Toner H, McWalter A, Eastwood J, Marshall M, Turvey T (1998). A community needs assessment: the care needs assessment pack for dementia (CarenapD)--its development, reliability and validity. Int J Geriatr Psychiatry.

[30] Sadak T, Korpak A, Borson S (2015). Measuring caregiver activation for health care: Validation of PBH-LCI:D. Geriatr Nurs.

[31] Pope C, Ziebland S, Mays N (2000). Qualitative research in health care. Analysing qualitative data. BMJ.

[32] Pope C, Mays N (1995). Reaching the parts other methods cannot reach: an introduction to qualitative methods in health and health services research. BMJ.

[33] Sörensen S, Pinquart M, Duberstein P (2002). How effective are interventions with caregivers? An updated meta-analysis. The Gerontologist.

[34] Pinquart M, Sörensen S (2006). Helping caregivers of persons with dementia: which interventions work and how large are their effects?. Int Psychogeriatr.

[35] Selwood A, Johnston K, Katona C, Lyketsos C, Livingston G (2007). Systematic review of the effect of psychological interventions on family caregivers of people with dementia. J Affect Disord.

[36] Thompson CA, Spilsbury K, Hall J, Birks Y, Barnes C, Adamson J (2007). Systematic review of information and support interventions for caregivers of people with dementia. BMC Geriatr.

[37] Aminzadeh F, Byszewski A, Dalziel WB, Wilson M, Deane N, Papahariss-Wright S (2005). Effectiveness of outpatient geriatric assessment programs: exploring caregiver needs, goals, and outcomes. J Gerontol Nurs.

[38] Johnston D, Samus QM, Morrison A, Leoutsakos JS, Hicks K, Handel S (2011). Identification of community-residing individuals with dementia and their unmet needs for care. Int J Geriatr Psychiatry.

[39] Black BS, Johnston D, Morrison A, Rabins PV, Lyketsos CG, Samus QM (2012). Quality of life of community-residing persons with dementia based on self-rated and caregiver-rated measures. Qual Life Res.

[40] Wackerbarth SB, Johnson MMS (2002). Essential information and support needs of family caregivers. Patient Educ Couns.

[41] Simonton L (1987). Assessing Caregiver Information Needs. J Gerontol Soc Work.

[42] Fortinsky RH, Hathaway TJ (1990). Information and service needs among active and former family caregivers of persons with Alzheimer’s disease. The Gerontologist.

[43] Francis GM, Munjas BA (1992). Needs of family caregivers and persons with Alzheimer’s disease. Am J Alzheimers Dis Other Demen.

[44] George LK, Fillenbaum GG (1985). OARS methodology. A decade of experience in geriatric assessment. J Am Geriatr Soc.

[45] Bowd AD, Loos CH (1996). Needs, morale and coping strategies of caregivers for persons with Alzheimer’s disease in isolated communities in Canada. Am J Alzheimers Dis Other Demen.

[46] Edelman P, Kuhn D, Fulton BR, Kyrouac GA (2006). Information and service needs of persons with Alzheimer’s disease and their family caregivers living in rural communities. Am J Alzheimers Dis Other Demen.

[47] Raivio M, Eloniemi-Sulkava U, Laakkonen M-L, Saarenheimo M, Pietilä M, Tilvis R (2007). How do officially organized services meet the needs of elderly caregivers and their spouses with Alzheimer’s disease?. Am J Alzheimers Dis Other Demen.

[48] Coudin G, Mollard J (2011). Difficulties, coping strategies and satisfactions in family caregivers of people with Alzheimer’s disease. Geriatr Psychol Neuropsychiatr Vieil.

[49] McKee K, Spazzafumo L, Nolan M, Wojszel B, Lamura G, Bien B (2009). Components of the difficulties, satisfactions and management strategies of carers of older people: a principal component analysis of CADI-CASI-CAMI. Aging Ment Health.

[50] Amieva H, Rullier L, Bouisson J, Dartigues J-F, Dubois O, Salamon R (2012). Needs and expectations of Alzheimer’s disease family caregivers. Rev Epidemiol Sante Publique.

[51] Laprise R, Dufort F, Lavoie F (2001). Construction et validation d’une echelle d’attentes en matiere de consultation aupres d’aidant (e) s de personnes agees. Can J Aging.

[52] Chow TW, Pio FJ, Rockwood K (2011). An international needs assessment of caregivers for frontotemporal dementia. Can J Neurol Sci.

[53] Diehl-Schmid J, Schmidt E-M, Nunnemann S, Riedl L, Kurz A, Förstl H (2013). Caregiver burden and needs in frontotemporal dementia. J Geriatr Psychiatry Neurol.

[54] Nicolaou PL, Egan SJ, Gasson N, Kane RT (2010). Identifying needs, burden, and distress of carers of people with Frontotemporal dementia compared to Alzheimer’s disease. Dementia.

[55] van der Roest HG, Meiland FJM, van Hout HPJ, Jonker C, Dröes R-M (2008). Validity and reliability of the Dutch version of the Camberwell Assessment of Need for the Elderly in community-dwelling people with dementia. Int Psychogeriatr.

[56] Galvin JE, Duda JE, Kaufer DI, Lippa CF, Taylor A, Zarit SH (2010). Lewy body dementia: caregiver burden and unmet needs. Alzheimer Dis Assoc Disord.

[57] Killen A, Flynn D, De Brún A, O’Brien N, O’Brien J, Thomas AJ (2016). Support and information needs following a diagnosis of dementia with Lewy bodies. Int Psychogeriatr.

[58] England M (2001). Expressed Information and Resource Needs of Filial Caregivers Reporting Recent Experiences of Crisis. Educ Gerontol.

[59] Gibson AK, Anderson KA, Acocks S (2014). Exploring the service and support needs of families with early-onset Alzheimer’s disease. Am J Alzheimers Dis Other Demen.

[60] Philp I, McKee KJ, Meldrum P, Ballinger BR, Gilhooly ML, Gordon DS (1995). Community care for demented and non-demented elderly people: a comparison study of financial burden, service use, and unmet needs in family supporters. BMJ.

[61] Turner SA, Street HP (1999). Assessing carers’ training needs: A pilot inquiry. Aging Ment Health.

[62] Gaugler JE, Anderson KA, Leach MSWCR, Smith CD, Schmitt FA, Mendiondo M (2004). The emotional ramifications of unmet need in dementia caregiving. Am J Alzheimers Dis Other Demen.

[63] Orrell M, Hancock GA, Liyanage KCG, Woods B, Challis D, Hoe J (2008). The needs of people with dementia in care homes: the perspectives of users, staff and family caregivers. Int Psychogeriatr.

[64] Selwood A, Cooper C, Owens C, Blanchard M, Livingston G (2009). What would help me stop abusing? The family carer’s perspective. Int Psychogeriatr.

[65] Nichols LO, Martindale-Adams J, Greene WA, Burns R, Graney MJ, Lummus A (2008). Dementia Caregivers’ Most Pressing Concerns. Clin Gerontol.

[66] Peeters JM, Van Beek AP, Meerveld JH, Spreeuwenberg PM, Francke AL (2010). Informal caregivers of persons with dementia, their use of and needs for specific professional support: a survey of the National Dementia Programme. BMC Nurs.

[67] Koenig KN, Steiner V, Pierce LL (2011). Information needs of family caregivers of persons with cognitive versus physical deficits. Gerontol Geriatr Educ.

[68] Li H (2012). Unmet service needs: a comparison between dementia and non-dementia caregivers. Home Health Care Serv Q.

[69] Miranda-Castillo C, Woods B, Orrell M (2013). The needs of people with dementia living at home from user, caregiver and professional perspectives: a cross-sectional survey. BMC Health Serv Res.

[70] Zwaanswijk M, Peeters JM, van Beek AP, Meerveld JH, Francke AL (2013). Informal caregivers of people with dementia: problems, needs and support in the initial stage and in subsequent stages of dementia: a questionnaire survey. Open Nurs J.

[71] Beisecker AE, Chrisman SK, Wright LJ (1997). Perceptions of family caregivers of persons with Alzheimer’s disease: Communication with physicians. Am J Alzheimers Dis Other Demen..

[72] Loukissa D, Farran CJ, Graham KL (1999). Caring for a relative with Alzheimer’s disease: The experience of African-American and Caucasian caregivers. Am J Alzheimers Dis Other Demen.

[73] Smith AL, Lauret R, Peery A, Mueller T (2001). Caregiver Needs. Clin Gerontol.

[74] Farran CJ, Loukissa D, Perraud S, Paun O (2003). Alzheimer’s disease caregiving information and skills. Part I: care recipient issues and concerns. Res Nurs Health.

[75] Farran CJ, Loukissa D, Perraud S, Paun O (2004). Alzheimer’s disease caregiving information and skills. Part II: family caregiver issues and concerns. Res Nurs Health.

[76] Nichols KR, Fam D, Cook C, Pearce M, Elliot G, Baago S (2013). When dementia is in the house: needs assessment survey for young caregivers. Can J Neurol Sci.

[77] Bakker C, de Vugt ME, Vernooij-Dassen M, van Vliet D, Verhey FRJ, Koopmans RTCM (2010). Needs in early onset dementia: A qualitative case from the NeedYD study. Am J Alzheimers Dis Other Demen.

[78] Millenaar JK, van Vliet D, Bakker C, Vernooij-Dassen MJFJ, Koopmans RTCM, Verhey FRJ (2014). The experiences and needs of children living with a parent with young onset dementia: results from the NeedYD study. Int Psychogeriatr.

[79] Boots LMM, Wolfs CAG, Verhey FRJ, Kempen GIJM, de Vugt ME (2015). Qualitative study on needs and wishes of early-stage dementia caregivers: the paradox between needing and accepting help. Int Psychogeriatr.

[80] Wawrziczny E, Pasquier F, Ducharme F, Kergoat M-J, Antoine P. Do spouse caregivers of young and older persons with dementia have different needs? A comparative study. Psychogeriatrics. 2017. In press. 10.1111/psyg.12234 28130806

[81] Bråne G (1986). Normal aging and dementia disorders—coping and crisis in the family. Prog Neuro-Psychopharmacol Biol Psychiatry.

[82] Lampley-Dallas VT, Mold JW, Flori DE (2001). Perceived needs of African-American caregivers of elders with dementia. J Natl Med Assoc.

[83] Shaji KS, Smitha K, Lal KP, Prince MJ (2003). Caregivers of people with Alzheimer’s disease: a qualitative study from the Indian 10/66 Dementia Research Network. Int J Geriatr Psychiatry.

[84] Innes A, Blackstock K, Mason A, Smith A, Cox S (2005). Dementia care provision in rural Scotland: service users’ and carers’ experiences. Health Soc Care Community.

[85] de Jong JD, Boersma F (2009). Dutch psychogeriatric day-care centers: a qualitative study of the needs and wishes of carers. Int Psychogeriatr.

[86] Shanley C, Russell C, Middleton H, Simpson-Young V (2011). Living through end-stage dementia: The experiences and expressed needs of family carers. Dementia.

[87] Samia LW, Hepburn K, Nichols L (2012). « Flying by the seat of our pants »: what dementia family caregivers want in an advanced caregiver training program. Res Nurs Health.

[88] Low L-F, White F, Jeon Y-H, Gresham M, Brodaty H (2013). Desired characteristics and outcomes of community care services for persons with dementia: what is important according to clients, service providers and policy?. Australas J Ageing.

[89] Vaingankar JA, Subramaniam M, Picco L, Eng GK, Shafie S, Sambasivam R (2013). Perceived unmet needs of informal caregivers of people with dementia in Singapore. Int Psychogeriatr.

[90] Muders P, Zahrt-Omar CA, Bussmann S, Haberstroh J, Weber M (2015). Support for families of patients dying with dementia: a qualitative analysis of bereaved family members’ experiences and suggestions. Palliat Support Care.

[91] Meyer OL, Nguyen KH, Dao TN, Vu P, Arean P, Hinton L (2015). The sociocultural context of caregiving experiences for Vietnamese dementia family caregivers. Asian Am J Psychol.

[92] Griffiths J, Bunrayong W (2016). Problems and needs in helping older people with dementia with daily activities: Perspectives of Thai caregivers. Br J Occup Ther.

[93] Peterson K, Hahn H, Lee AJ, Madison CA, Atri A (2016). In the Information Age, do dementia caregivers get the information they need? Semi-structured interviews to determine informal caregivers’ education needs, barriers, and preferences. BMC Geriatr.

[94] Samson ZB, Parker M, Dye C, Hepburn K (2016). Experiences and Learning Needs of African American Family Dementia Caregivers. Am J Alzheimers Dis Other Demen.

[95] Jennings LA, Palimaru A, Corona MG, Cagigas XE, Ramirez KD, Zhao T, et al. Patient and caregiver goals for dementia care. Qual Life Res. 2016;26(3):685–93 10.1007/s11136-016-1471-7 PMC5382930 28000094

[96] Wolfs CAG, de Vugt ME, Verkaaik M, Verkade P-J, Verhey FRJ (2010). Empowered or overpowered? Service use, needs, wants and demands in elderly patients with cognitive impairments. Int J Geriatr Psychiatry.

[97] Kuhn DR (1998). Caring for relatives with early stage Alzheimer’s disease: An exploratory study. Am J Alzheimers Dis Other Demen.

[98] Habermann B, Davis LL (2005). Caring for family with Alzheimer’s disease and Parkinson’s disease: needs, challenges and satisfaction. J Gerontol Nurs.

[99] Ducharme F, Kergoat M-J, Coulombe R, Lévesque L, Antoine P, Pasquier F (2014). Unmet support needs of early-onset dementia family caregivers: a mixed-design study. BMC Nurs.

[100] Leong J, Madjar I, Fiveash B (2001). Needs of Family Carers of Elderly People with Dementia Living in the Community. Australas J Ageing.

[101] Stirling C, Andrews S, Croft T, Vickers J, Turner P, Robinson A (2010). Measuring dementia carers’ unmet need for services--an exploratory mixed method study. BMC Health Serv Res.

