# Supplementary material for: How to explore the needs of informal caregivers of individuals with cognitive impairment in Alzheimer’s disease or related diseases? A systematic review of quantitative and qualitative studies
Source: BMC Geriatr. 2017 Apr 17;17:86. doi: 10.1186/s12877-017-0481-9 (PMC5393006; doi:10.1186/s12877-017-0481-9)
Supplement: Additional file 1: — Search strategies. This file provide the search strategies used in MEDLINE, PsycINFO, The Cochrane Library and Web of Science in order to identify quantitative and qualitative studies for the systematic review. (DOC 25 kb) [file 12877_2017_481_MOESM1_ESM.doc]

**Additional file 1. Search strategies**

**MEDLINE (PubMed) < 1980 to present >**

1. carer* OR caregiver* OR loved one* OR family OR families

2. dementia OR Alzheimer* OR frontotemporal OR lewy OR vascular dementia OR cognitive impairment OR memory

3. need*

4. french""[Language]) OR ""english""[Language]"

5. quantitative* OR qualitative* OR questionnaire* OR item* OR scale* OR tool* OR instrument* OR interview* OR cross-sectional OR focus group* OR structured OR verbatim* OR survey

6. 1 AND 2 AND 3 AND 4 AND 5

**PsycINFO < 1980 to present >**

(Any Field: (quantitative) OR Any Field: (qualitative) OR Any Field: (survey) OR Any Field: (questionnaire) OR Any Field: (item) OR Any Field: (tool) OR Any Field: (instrument) OR Any Field: (focus group) OR Any Field: (interview) OR Any Field: (cross-sectional)) AND (Any Field: (dementia) OR Any Field: (alzheimer*) OR Any Field: (frontotemporal) OR Any Field: (lewy) OR Any Field: (vascular dementia) OR Any Field: (memory) OR Any Field: (cognitive impairment) OR Any Field: (cognitive complaint)) AND (Any Field: (need*) OR Any Field: (expectation*)) AND (Any Field: (caregiver*) OR Any Field: (carer*) OR Any Field: (famil*) OR Any Field: (loved one*)) AND (Language:(french) OR Language:(english))

**The Cochrane Library < 1980 to present >**

1. need*:ti,ab,kw

2. caregiver*:ti,ab,kw or carer*:ti,ab,kw or loved one*:ti,ab,kw or famil*:ti,ab,kw

3. dementia:ti,ab,kw or Alzheimer* or “frontotemporal” or lewy* or “vascular dementia”

4. 1 and 2 and 3

5. “quantitative” :ti,ab,kw or “survey” :ti,ab,kw or “cross-sectional” :ti,ab,kw or instrument:ti,ab,kw or questionnaire:ti,ab,kw

6. “qualitative” :ti,ab,kw or “focus group” :ti,ab,kw or “interview” :ti,ab,kw or “structured” :ti,ab,kw or tool*:ti,ab,kw

7. 5 or 6

8. 4 and 7

**Web of Science < 1980 to present >, search language = French and English**

1. TS=(caregiver* OR carer* OR loved one* OR famil*)

2. TS=(quantitative OR qualitative OR survey OR questionnaire OR item OR tool OR instrument OR focus group OR interview or Cross sectional)

3. TS=(dementia OR Alzheimer* OR frontotemporal OR lewy OR vascular dementia OR memory OR cognitive impairment OR cognitive complaint)

4. TS=(need* OR expectation*)

5. 4 AND 3 AND 2 AND 1
